# Paediatric malaria case-management with artemether-lumefantrine in Zambia: a repeat cross-sectional study

**DOI:** 10.1186/1475-2875-6-31

**Published:** 2007-03-16

**Authors:** Dejan Zurovac, Mickey Ndhlovu, Nawa Sipilanyambe, Pascalina Chanda, Davidson H Hamer, Jon L Simon, Robert W Snow

**Affiliations:** 1 Malaria Public Health and Epidemiology Group, Centre for Geographic Medicine, KEMRI/Wellcome Trust Collaborative Programme, P.O. Box 43640, 00100 GPO, Nairobi, Kenya; 2 Centre for Tropical Medicine, University of Oxford, John Radcliffe Hospital, Headington, Oxford OX3 9DU, UK; 3 Chainama Hills College Hospital of Health Sciences, P.O. Box 33991, Lusaka, Zambia; 4 National Malaria Control Centre, Ministry of Health, P.O. Box 32509, Lusaka, Zambia; 5 Center for International Health and Development, Boston University School of Public Health, 85 East Concord Street, 5^th ^Floor, Boston, MA 02118, USA; 6 Section of Infectious Diseases, Department of Medicine, Boston University School of Medicine, Boston, MA, 02118, USA

## Abstract

**Background:**

Zambia was the first African country to change national antimalarial treatment policy to artemisinin-based combination therapy – artemether-lumefantrine. An evaluation during the early implementation phase revealed low readiness of health facilities and health workers to deliver artemether-lumefantrine, and worryingly suboptimal treatment practices. Improvements in the case-management of uncomplicated malaria two years after the initial evaluation and three years after the change of policy in Zambia are reported.

**Methods:**

Data collected during the health facility surveys undertaken in 2004 and 2006 at all outpatient departments of government and mission facilities in four Zambian districts were analysed. The surveys were cross-sectional, using a range of quality of care assessment methods. The main outcome measures were changes in health facility and health worker readiness to deliver artemether-lumefantrine, and changes in case-management practices for children below five years of age presenting with uncomplicated malaria as defined by national guidelines.

**Results:**

In 2004, 94 health facilities, 103 health workers and 944 consultations for children with uncomplicated malaria were evaluated. In 2006, 104 facilities, 135 health workers and 1125 consultations were evaluated using the same criteria of selection. Health facility and health worker readiness improved from 2004 to 2006: availability of artemether-lumefantrine from 51% (48/94) to 60% (62/104), presence of artemether-lumefantrine dosage wall charts from 20% (19/94) to 75% (78/104), possession of guidelines from 58% (60/103) to 92% (124/135), and provision of in-service training from 25% (26/103) to 41% (55/135). The proportions of children with uncomplicated malaria treated with artemether-lumefantrine also increased from 2004 to 2006: from 1% (6/527) to 27% (149/552) in children weighing 5 to 9 kg, and from 11% (42/394) to 42% (231/547) in children weighing 10 kg or more. In both weight groups and both years, 22% (441/2020) of children with uncomplicated malaria were not prescribed any antimalarial drug.

**Conclusion:**

Although significant improvements in malaria case-management have occurred over two years in Zambia, the quality of treatment provided at the point of care is not yet optimal. Strengthening weak health systems and improving the delivery of effective interventions should remain high priority in all countries implementing new treatment policies for malaria.

## Background

By June 2006, 39 African countries had changed their policies to recommend artemisinin-based combination therapy (ACT) as the first line treatment for malaria [1] to reduce the devastating effects of failing monotherapies [2] and limit the spread of drug resistance [3]. This represents one of the most significant public health developments in malaria control for decades. To implement these policies, several key challenges remain: increasing sustainable financing of these expensive treatments, improving prompt access through better care-seeking at health facilities and high quality community-based delivery systems, and ensuring these new drugs are used appropriately during clinical management.

Of these challenges, inadequate case-management practices are of particular concern. In health facilities across Africa, febrile children are often treated suboptimally [4-7], incorrect doses of antimalarials are frequently prescribed [8-10], and appropriate counseling and drug dispensing is rarely provided [10-12]. Therefore, the introduction of ACTs, which are new, expensive and more complex antimalarial regimens with less well described safety profiles poses, both a challenge and an opportunity to the quality of malaria management in Africa.

In 2002, due to chloroquine resistance [13-15], Zambia became one of the first countries in Africa to change its treatment policy for uncomplicated malaria from chloroquine to ACT: artemether-lumefantrine for children and non-pregnant adults weighing 10 kg or more and sulphadoxine-pyrimethamine for children less than 10 kg [16]. The Zambian Government, with support from external funding partners, made substantial progress in 2003 and 2004 to secure adequate drug supplies, conduct in-service training for health workers, and revise clinical guidelines and wall charts [16].

Health workers' performance in treating febrile paediatric patients at government clinics in Zambia was reported when the new drug policy was in its nascent implementation phase [17]. Here, a follow-up study was presented to evaluate how febrile children are managed with artemether-lumefantrine approximately two years after the initial survey.

## Methods

### Study sites and timing

The survey was undertaken in four districts purposively selected to represent major malaria ecologies in Zambia: Chingola, Kalomo, Chipata and Samfya [17]. All government and mission health facilities providing outpatient care were surveyed between January and March 2004 to assess the quality of malaria case-management, three to 11 months following the implementation of the new artemether-lumefantrine drug policy. Between March and May 2006, a follow-up survey was undertaken at all facilities in the same districts. Ethical approval was provided by the Boston University IRB (2003-412B and H-25346) and Research Ethics Committee of the University of Zambia (Federal Wide Assurance Number IRB00001131 and IRB00000338).

### Survey design and data collection

The quality of antimalarial prescriptions, counseling and drug dispensing was studied using a cross sectional, cluster sample survey at all health facilities in four districts using similar methods in both the 2004 and 2006 surveys. A cluster was defined as all sick outpatients seen at a health facility during one working day. Each facility was randomly assigned to one survey day and all patients presenting to outpatient departments during the survey day were recruited. Here, case-management practices in children below five years of age were described, while data on patients five years and above will be presented elsewhere.

Data were collected by four teams, each composed of two surveyors, using three methods: 1) exit interviews with caretakers, 2) health worker interviews, and 3) health facility assessments. Prior to the interviews, all caretakers and health workers were asked to provide written informed consent. For exit interviews, all caretakers of sick children were interviewed when they completed the health facility visit. Interviewers asked questions about the child's age, history of fever during the present illness, use of antimalarial drugs prior to the facility visit, and if the visit was an initial or follow-up consultation. Information was also collected from patient held records about routine diagnostic procedures requested and results reported, medications prescribed, and if the child was treated as an outpatient or referred for hospitalisation. For prescribed antimalarial drugs, interviewers assessed if the drug was dispensed, whether swallowing of the first dose was observed at the health facility, and if the caretaker received instructions on how to give the drugs at home. This information was obtained from caretaker interviews and referred not only to the consultation but also to any time during the facility visit. Finally, during the exit interview, each child was weighed and the axillary temperature was measured.

Health worker interviews were conducted after working hours with all health workers who had attended sick children during the survey day. Health workers were asked about their demographics, pre-service training, working experience, supervisory visits, possession of guidelines, and exposure to in-service case-management trainings. Health workers' knowledge about recommended first-line treatment for various patient groups with uncomplicated malaria was assessed using open-ended questions.

Finally, a health facility assessment was performed to record the availability of medical supplies and equipment related to malaria case-management. The presence of weighing scales, thermometers, malaria treatment wall charts and malaria diagnostics (microscopy and rapid diagnostic tests) was assessed. Particular emphasis was paid to the availability of antimalarial drugs on the day of the survey and stock-out periods in the past 12 months.

### Definitions

Diagnosis definitions reflected recommendations from Zambian national guidelines [18,19] and training manuals used during in-service training for health workers on malaria case-management [20,21]. According to these reference materials, any child with fever or history of fever in the absence of signs of severe malaria was presumed to have uncomplicated malaria in high malaria risk areas. For clinical management purposes, all Zambia is considered as a high malaria risk area and the presence of fever in children irrespective of other signs provides enough evidence to suspect malaria [19-21]. A case of uncomplicated malaria was defined as a child below five years of age who presented to a health facility for an initial visit with a history of fever during the present illness or an axillary temperature of 37.5°C or more, and who was treated as an outpatient in the absence of a negative malaria test.

Ambiguities exist regarding the role of malaria diagnostics in malaria case-management. The national malaria guideline states that the "presence of signs and symptoms of disease with negative blood smear does not preclude the diagnosis of malaria" [18] and similar ambiguous recommendations appear in training manuals [20,21]. Regarding the interpretation of rapid diagnostic tests, none of the reference materials provides instructions on how health workers should act upon negative results. To prevent health worker practices from being judged as incorrect due to ambiguity, children with a negative malaria test were excluded from the analysis.

Recommended treatment for uncomplicated malaria was defined according to national guidelines and training materials: artemether-lumefantrine for children weighing 10 kg or more and sulphadoxine-pyrimethamine for children less than 10 kg [18-23]. Further ambiguities that were subsequently identified in the latter recommendation are described in the results.

### Data entry and statistical analysis

Data were double-entered and verified in Microsoft Access and used STATA 8 (StataCorp, College Station, Texas) for statistical analysis. Descriptive analysis was undertaken at health facility, health worker and child level. At health facility and health worker level chi-square test was used to test significant differences in proportions between 2004 and 2006 surveys. At child level the precision of proportions (95% confidence interval) was estimated accounting for the cluster sampling design using "health facility-day" as the primary sampling unit. Hypothesis testing and confidence interval estimation were done with an alpha level of 0.05.

## Results

### Changes in health facility and health worker readiness to deliver artemether-lumefantrine

There were 94 facilities assessed in 2004 and 104 in 2006 (table 1). The distribution of health facility types was similar between the two surveys. The presence of thermometers and weighing scales was generally high in both survey rounds. However in June 2005 the introduction of malaria rapid diagnostic tests into the facilities greatly increased parasitological diagnostic capacity, from 17% (16/94) in 2004 based solely on microscopy to 73% (76/104) (P < 0.001) in 2006 largely relying on rapid diagnostic tests. The widespread distribution of revised artemether-lumefantrine dosing wall charts from November 2005 also greatly increased the availability of these job aides, from 20% (19/94) in 2004 to 75% (78/104) (P < 0.001) in 2006.

**Table 1:** Characteristics of health facilities and health workers during 2004 and 2006 surveys in Zambia

	**2004**	**2006**
**Health facility characteristics**	N = 94	N = 104
	No (%)	No (%)

Type of facility		
Hospital-affiliated health center	7 (7.4)	6 (5.8)
Urban health center	7 (7.4)	8 (7.7)
Rural health center	78 (83.0)	87 (83.7)
Health post	2 (2.1)	3 (2.9)
Equipment available at facility		
Weighing scale	92 (97.9)	99 (97.1)*
Thermometer^†^	91 (97.8)^‡^	90 (88.2)*
Working microscope	16 (17.0)	18 (17.3)
Malaria rapid diagnostic test^†^	0	65 (62.5)
Any test (malaria rapid diagnostic test or microscopy)^†^	16 (17.0)	76 (73.1)
Wall charts		
Artemether-lumefantrine dosage chart^†^	19 (20.2)	78 (75.0)
Sulphadoxine-pyrimethamine dosage chart	48 (51.1)	39 (37.5)
Quinine dosage chart	4 (4.3)	4 (3.9)
Available drugs on the survey day		
Artemether-lumefantrine (any non-expired tablets)	48 (51.1)	62 (59.6)
Sulphadoxine-pyrimethamine (any formulation)^†^	94 (100)	96 (92.3)
Quinine (injection)	68 (72.3)	78 (75.0)
Quinine (tablets)	74 (78.7)	90 (86.5)
Chloroquine (any formulation)^†^	71 (75.5)	0

**Health worker characteristics**	N = 103	N = 135
	No (%)	No (%)

Pre-service training		
Clinical officer	30 (29.1)	38 (28.2)^§^
Enrolled Nurse	49 (47.6)	52 (38.5)
Registered nurse	4 (3.9)	10 (7.4)
Other	20 (19.4)	35 (25.9)
In-service training including recommendations on the use of artemether-lumefantrine^†^	26 (25.2)	55 (40.7)
Possession of guidelines that included artemether-lumefantrine recommendations^†^	60 (58.3)	124 (91.9)
Frequency of supervision (past 6 months)		
0 visit	14 (13.6)	16 (11.9)
1 visit^†^	22 (21.4)	56 (41.5)
2 visits	42 (40.8)	52 (38.5)
3 or more visits^†^	25 (24.3)	11 (8.1)

* Denominator does not include two observations with missing value

^† ^P-value < 0.05 using chi-square test of significance

^‡ ^Denominator does not include one observation with missing value

^§ ^The clinical officer category includes one physician

Chloroquine had been successfully removed from the health facilities by 2006 (table 1). Sulphadoxine-pyrimethamine was widely available in 2004 and 2006. Artemether-lumefantrine was available at 60% (62/104) of the facilities surveyed in 2006, a slight, though statistically not significant improvement over 2004 (51%, 48/94) (P = 0.227). During the 2006 survey, 12 facilities had incomplete stock-out documentation over the preceding 12 months. Among the remaining 92 facilities, artemether-lumefantrine stock-outs were common; on average health facilities experienced stock-outs of the different dose pack sizes ranging between 108 and 123 days. Seven percent (7/104) of facilities had expired stocks of artemether-lumefantrine.

A variety of in-service training activities had been undertaken since the launch of the new artemether-lumefantrine drug policy in Zambia and after the 2004 survey. Notably these included a national malaria training course sponsored by Novartis Pharma Ltd, provincial and district level trainings organized by the National Malaria Control Centre and the Integrated Management of Childhood Illnesses (IMCI) programme in Zambia began to train health workers on the use of artemether-lumefantrine. The proportion of health workers receiving any form of training that included artemether-lumefantrine rose from 25% (26/103) in 2004 to 41% (55/135) two years later (P < 0.001) (Table 1).

During the 2004 survey 58% (60/103) of health workers had been provided with a draft version of new national guideline on malaria case-management. These guidelines were officially printed and distributed to health workers from August 2004 [18]. Furthermore, new treatment recommendations for malaria have been gradually incorporated into IMCI guidelines [19], Integrated Technical Guidelines for Front Line Health Workers [22] and Standard Treatment Guidelines [23]. In 2006, the proportion of health workers who had in their possession at least one guideline that recommended use of artemether-lumefantrine rose to 92% (124/135) (P < 0.001). Similar proportions of health workers had received at least one supervisory visit in the six months prior to both the 2004 (86%, 89/103) and 2006 (88%, 119/135) surveys. During the six months preceding the 2006 survey, 71% (94/133; two missing values) of health workers reported at least one supervisory visit that included discussion on appropriate use of artemether-lumefantrine. Health workers' knowledge on the recommended treatment for uncomplicated malaria was generally high for children weighing 10 kg and more (87%, 117/135), however 33% (44/135) of health workers reported that artemether-lumefantrine was recommended for children weighing less than 10 kg.

### Changes in antimalarial treatment practices for children with uncomplicated malaria

During the 2006 survey, 1,498 children aged less than five years were evaluated as they left the clinic with their caretakers and were not referred for hospitalization. Four children were excluded from analysis because data were incomplete. Further 369 children were excluded because they presented for follow-up visit, had neither history of fever nor an axillary temperature of 37.5°C or more, or had a negative parasitological test. The influence of diagnostics on malaria diagnosis and treatment will be presented elsewhere. The remaining 1,125 children fulfilled our definition of uncomplicated malaria. Of these 1,125 children, 26 children weighed less than 5 kg, 552 were between 5 and 9 kg and 547 were 10 kg or more; comparable numbers of children were evaluated following the same selection criteria in 2004 (23, 527, and 394, respectively).

In 2004, during the early implementation of the treatment policy, artemether-lumefantrine was not recommended for children less than 10 kg; they were supposed to be treated with sulphadoxine-pyrimethamine. In October 2005, the Zambian Central Board of Health officially recommended the use of artemether-lumefantrine in patients weighing 5 to 9 kg [24]; and from November 2005, revised wall charts were distributed to health facilities that recommended that children weighing between 5 to 9 kg should be treated with six tablets of artemether-lumefantrine over three days. Given this discrepancy between the guidelines, primary analysis included only patients fulfilling our inclusion criteria who weighed 5 kg and more during both the 2004 and 2006 surveys and results are presented for children weighing 5 to 9 kg, and 10 kg or more separately. Of the 23 patients weighing less than 5 kg in 2004, six were not provided with an antimalarial, and 17 were prescribed sulphadoxine-pyrimethamine. In 2006, of the 26 patients weighing less than 5 kg, 14 did not receive an antimalarial, 10 received sulphadoxine-pyrimethamine and two were prescribed artemether-lumefantrine.

The treatment quality was first examined at all health facilities surveyed in 2004 and 2006, whether or not they had artemether-lumefantrine in stock (Table 2). In 2004, children with uncomplicated malaria weighing 5 to 9 kg were predominantly prescribed sulphadoxine-pyrimethamine (80%, 422/527), and only six children (1%) received artemether-lumefantrine. In 2006, more children in the same weight group were treated with artemether-lumefantrine (27%, 149/552), and 39% received sulphadoxine-pyrimethamine. Among children 10 kg or more, for whom the wall charts and guidelines are less ambiguous, the proportion of children receiving artemether-lumefantrine increased significantly from 11% (42/394) in 2004 to 42% (231/547) in 2006 (table 2).

**Table 2:** Antimalarial treatments for children with uncomplicated malaria presenting to all health facilities – change of practice between 2004 and 2006 surveys in four districts in Zambia

**2004**	**5–9 kg (N = 527)**		**≥ 10 kg (N = 394)**	
	
	**No (%)**	95% CI*	**No (%)**	95% CI*
Artemether-lumefantrine	6 (1.1)	0, 2.7	42 (10.7)	5.4, 16.0
Sulphadoxine-pyrimethamine	422 (80.1)	73.9, 86.2	266 (67.5)	58.8, 76.2
Quinine	14 (2.7)	0.8, 4.5	19 (4.8)	2.0, 7.6
Chloroquine	1 (0.2)	0, 0.6	0	NA
No antimalarial prescribed	84 (15.9)	10.3, 21.6	67 (17.0)	11.7, 22.3

**2006**	**5–9 kg (N = 552)**		**≥ 10 kg (N = 547)**	
	
	**No (%)**	95% CI*	**No (%)**	95% CI*

Artemether-lumefantrine	149 (27.0)	19.2, 34.8	231 (42.2)	33.8, 50.7
Sulphadoxine-pyrimethamine	214 (38.8)	30.9, 46.7	151 (27.6)	19.9, 35.3
Quinine	26 (4.7)	1.5, 7.9	38 (7.0)	2.5, 11.4
No antimalarial prescribed	163 (29.5)	23.3, 35.8	127 (23.2)	17.9, 28.5

* Confidence intervals adjusted for cluster sampling

Many children with uncomplicated malaria left clinics without any antimalarial prescribed: 22% (441/2020) across both weight groups and both years (Table 2). In 74% (327/441) of these children, a diagnosis of unspecified respiratory tract infection (30%, 131/441), pneumonia (16%, 70/441), eye infection (15%, 68/441), diarrhoeal disease (12%, 52/441), skin infection (11%, 50/441) or ear infection (3%, 14/441) was recorded. Conversely, only 34% (534/1579) of children having an antimalarial drug prescribed had any of these diagnoses recorded.

Of particular interest during the 2004 survey was the observation that only 22% (42/192) of children with uncomplicated malaria weighing 10 kg or more who presented to a facility where artemether-lumefantrine was in stock on the day of the survey received artemether-lumefantrine and 54% (103/192) of these children were prescribed sulphadoxine-pyrimethamine (table 3). Two years later the proportion of children prescribed artemether-lumefantrine rose significantly to 59% (219/374). This was also true for the increase in artemether-lumefantrine prescriptions for children weighing 5 to 9 kg, which rose from 2% (6/254) in 2004 to 41% (144/351) in 2006. An important proportion (26%, 309/1,171) of children of any weight group in both years still left these clinics where artemether-lumefantrine was in stock without any antimalarial (Table 3).

**Table 3:** Antimalarial treatments for children with uncomplicated malaria presenting to health facilities with artemether-lumefantrine in stock – change of practice between 2004 and 2006 surveys in four districts in Zambia

**2004**	**5–9 kg (N = 254)**		**≥ 10 kg (N = 192)**	
	
	**No (%)**	95% CI*	**No (%)**	95% CI*
Artemether-lumefantrine	6 (2.4)	0, 5.7	42 (21.9)	12.4, 31.3
Sulphadoxine-pyrimethamine	183 (72.1)	61.0, 83.1	103 (53.7)	39.7, 67.6
Quinine	9 (3.5)	0.3, 6.8	6 (3.1)	0.4, 5.9
No antimalarial prescribed	56 (22.1)	11.6, 32.5	41 (21.4)	12.7, 30.0

**2006**	**5–9 kg (N = 351)**		**≥ 10 kg (N = 374)**	
	
	**No (%)**	95% CI*	**No (%)**	95% CI*

Artemether-lumefantrine	144 (41.0)	31.5, 50.5	219 (58.6)	50.2, 66.9
Sulphadoxine-pyrimethamine	77 (21.9)	15.4, 28.5	51 (13.6)	7.1, 20.2
Quinine	8 (2.3)	0.1, 4.5	14 (3.7)	0, 7.9
No antimalarial prescribed	122 (34.8)	27.2, 42.3	90 (24.1)	17.4, 30.7

* Confidence intervals adjusted for cluster sampling

### Artemether-lumefantrine dosage, drug dispensing, and counselling

The correctness of prescribed artemether-lumefantrine dosages was assessed according to weight-specific criteria. The correctness was very high (81% to 96%) in all weight groups, except for children 15–24 kg because they tended to receive dosages designed for a lower weight category (table 4). Of the 48 children having artemether-lumefantrine dispensed in 2004, 21 (44%) had their first dose administered at the facility, all under observation of health workers. In 2006, 58% (206/354) were given the first dose at the facility and nearly all (97%, 200/206) had swallowing of the first dose observed. All 48 caretakers were provided advice on the dosing schedule of artemether-lumefantrine in 2004 as were 98% (347/355) in 2006. Advice to take artemether-lumefantrine after the meal was provided to only 31% (15/48) of caretakers in 2004 but this rose to 71% (251/355) in 2006.

**Table 4:** Correctness of artemether-lumefantrine dosage prescriptions – change of practice between 2004 and 2006 surveys in four districts in Zambia

	**Recommended No (%)**	**Overdose No (%)**	**Underdose No (%)**	**Dose not specified No (%)**
**2004**				
All children 5–24 kg (N = 48)	39(81.3)	2 (4.2)	5 (10.4)	2 (4.2)
5–9 kg (N = 6)	5 (83.3)	1 (16.7)	0	0
10–14 kg (N = 34)	31 (91.2)	1 (2.9)	0	2 (5.9)
15–24 kg (N = 8)	3 (37.5)	0	5 (62.5)	0

**2006**				
All children 5–24 kg (N = 380)	338 (89.0)	11 (2.9)	28 (7.4)	3 (0.8)
5–9 kg (N = 149)	143 (96.0)	0	5 (3.4)	1 (0.7)
10–14 kg (N = 185)	173 (93.5)	10 (5.4)	1 (0.5)	1 (0.5)
15–24 kg (N = 46)	22 (47.8)	1 (2.2)	22 (47.8)	1 (2.2)

## Discussion

### Improvements in implementing artemether-lumefantrine policy

Programmatic activities improved the readiness of health facilities and health workers to deliver artemether-lumefantrine to febrile children between 2004 and 2006. Facilities with trained staff, guidelines and wall charts developed around the new drug policy had increased two years following the early implementation survey (Table 1). At facilities where artemether-lumefantrine was in stock, the proportions of children weighing 10 kg or more, who should have received this drug, increased from 22% in 2004 to 59% in 2006 (Table 3). Although results are not yet optimal, these are encouraging findings. However, they further demonstrate that the implementation of new treatment policies is a long process where even several years after the launch of the process further interventions are still required. This is an important lesson for other countries either starting to implement ACTs or which opted for interim treatment strategies while awaiting decisions on ACT policies.

### Challenges facing optimum implementation of artemether-lumefantrine policy

There continue to be several important health system and information dissemination problems that limit effective implementation of the new drug policy. First, drug stock management is probably a major rate-limiting step to effective delivery of artemether-lumefantrine. In 2006, 40% of facilities still did not have any artemether-lumefantrine in stock on the survey day, facilities were out of stock for approximately 30% of the year, and some (7%) had expired drugs. Ensuring adequate supplies of artemether-lumefantrine is a complex process in Zambia, critically dependent on the quantification of consumption for each of four different dose packages, processing of orders through districts to national stores, and subsequent prompt distribution of adequate quantities to districts and facilities. The effectiveness of the supply chain is further complicated with international procurement of artemether-lumefantrine, single source of the product and potential shortages of the drug on the global market. Further investigation of artemether-lumefantrine supply chain and strengthening of the drug distribution is an immediate priority in Zambia and probably in other countries implementing ACTs in Africa [25].

Second, the implementation process of artemether-lumefantrine was launched in 2003 when there was little published data and no international guidance, on the use of this drug among patients weighing less than 10 kg. The national guidelines therefore recommended that artemether-lumefantrine be withheld from patient's weighing less than 10 kg and that sulphadoxine-pyrimethamine should be used in this weight group. This clearly posed problems in Zambia, where there was evidence that sulphadoxine-pyrimethamine failure rates were already high [14,26]. In June 2005, Falade and colleagues published multi-site trial data among infants weighing 5 to 9 kg and reported that artemether-lumefantrine was both safe and efficacious in this patient group [27]. In October 2005, the Zambian Central Board of Health made an important decision to recommend artemether-lumefantrine in patients with uncomplicated malaria weighing 5 to 9 kg. This weight group recommendation is consistent with those specified as part of national guidelines developed during 2005 and 2006 in Kenya [28], Tanzania [29] and Uganda [30]. Wall charts and notification to health facility staff in Zambia were disseminated with these revised instructions toward the end of 2005. An inevitable consequence of this change was that national formulary, IMCI flowcharts and clinical guidelines revised between 2003 and 2005 around sulphadoxine-pyrimethamine recommendations for children less than 10 kg now differed from the latest recommendations. Furthermore, prior to this recent revision, health workers have been trained to use artemether-lumefantrine only in children 10 kg and above. Consequently fewer children 5 to 9 kg in 2006 had received this drug compared to children weighing 10 kg or more (Tables 2 and 3).

The early introduction of new effective post-registration drugs into public health use is important when current therapies are failing to cure life threatening illnesses like malaria. However, the results of trials in special groups often emerge post-registration and national health officials need to respond to any changing recommendations. Changing and implementing revisions to a major drug policy, such as the treatment of malaria, is fraught with financing, coordination and human resource problems that can overwhelm the government agencies charged with this responsibility [31,32]. Updating revisions to new treatment recommendations is critical but often a neglected dynamic component of policy implementation.

The Zambian studies in 2004 and 2006 show that artemether-lumefantrine dosing was largely accurate among the two dominant weight group categories of 5 to 9 kg and 10 to 14 kg (Table 4), and advice was given on how to take subsequent doses at home to the majority of caretakers. These are encouraging findings given the common dosing and counseling deficiencies reported for sulphadoxine-pyrimethamine use in Kenya [10]. However, an area of concern is that approximately one in five febrile children still left the clinic in both years without any antimalarial prescribed (Tables 2 and 3). Most of rural Zambia, including all our study districts, is located in high malaria risk areas regarded by national and international guidelines as meriting treatment of all childhood fevers with antimalarial drugs irrespective of other causes [18-21,33]. However, closer examination of the patient group that did not receive any antimalarial treatment revealed a variety of diagnoses made by the examining health workers. It appears that health workers were making a differential diagnosis, that led to a clinical judgment on other possible causes of the presenting fever and thus not presumptively treating all fevers as malaria as recommended in guidelines and training materials.

## Conclusion

The studies reported here provide important information to measure the operational implementation of new drug policies. The Zambian experience as an early implementer of large-scale use of ACT offers valuable lessons for both further improvements in the national program and also for other countries in the region. Though improvements in health facility and health worker readiness and appropriate case-management occurred during the initial three year's experience, major areas of suboptimal performance still exist – especially in ensuring the effectiveness of the drug supply and management, improving effective translation of research into guidelines, and subsequently guidelines into clinical practice. Furthermore, repeated evaluation research needs to become a regular part of program implementation with consistent financial support so program and global policies can learn in a timely fashion from the efforts currently underway to mitigate the mortality and morbidity impacts of the malaria scourge in Africa.

## Competing interests

DZ, NS and RWS have received a fee for speaking at a meeting organized by Novartis Pharma AG, the manufacturers of artemether-lumefantrine. MN, PC, DHH and JLS declared no competing interest.

## Authors' contributions

DZ contributed to the conception and design of the study, analysis and interpretation of results, and drafting/finalisation of the manuscript.

MN contributed to the conception and design of the study, analysis and interpretation of results, and drafting of the manuscript.

NS contributed to data analysis and interpretation of results, and drafting of the manuscript.

PC contributed to data analysis and interpretation of results, and drafting of the manuscript.

DHH contributed to the study design, data analysis and interpretation of results, and drafting of the manuscript.

JLS contributed to data analysis data analysis and interpretation of results, and drafting of the manuscript.

RWS contributed to the study design, data analysis and interpretation of results, and drafting/finalisation of the manuscript.
